# Continuous quality evaluation of the Asanté rapid test for recent infection for robust kit lot quality verification

**DOI:** 10.1371/journal.pgph.0003195

**Published:** 2024-05-14

**Authors:** Amy Zheng, Mervi Detorio, Trudy Dobbs, Vedapuri Shanmugam, Xiaojuan Tan, Jeni Vuong, Robert A. Domaoal, Kemba Lee, LaTasha Williams, Keisha Jackson, Bharat Parekh, Ernest L. Yufenyuy

**Affiliations:** 1 Public Health Institute/Centers for Disease Control Global Health Fellowship Program, Oakland, California, United States of America; 2 Division of Global HIV & Tuberculosis, Global Health Center, Centers for Disease Control and Prevention, Atlanta, Georgia, United States of America; University of Cape Town Faculty of Health Sciences, SOUTH AFRICA

## Abstract

The Sedia Biosciences Asanté rapid test for recent infection (RTRI) can identify HIV infections and characterize HIV-1 as recent or long-term infection via the positive verification (V) line and long-term line (LT) line, respectively. Tracking with Recency Assays to Control the Epidemic (TRACE) program uses RTRI assays. Successful implementation of TRACE requires high-quality test performance. The goal of this study is to evaluate the additional quality practices established for new kit lots prior to field use. Asanté lot quality control data from the manufacturer is reviewed by the Centers for Disease Control and Prevention International Laboratory Branch (CDC-ILB) in the Division of Global HIV and TB using. If a lot passes manufacturer quality control and CDC-ILB review, test kits are sent to CDC-ILB for further evaluation. Evaluation by CDC includes inter-rater reliability and linear regressions comparing the V and LT lines against reference data as well as V and LT line data between testers. A Bland-Altman analysis was conducted to assess bias and systematic error. Overall, CDC-ILB passed 29 (91%) out of 32 Sedia Biosciences Asanté kit lots that initially passed manufacturing quality control from July 2017 to May 2020. Regression analyses demonstrate that test kits are performing as expected with consistent R^2^≥0.92 for both V and LT lines. On average, inter-rater reliability kappa was 0.9, indicating a strong level of agreement. Bland-Altman analyses demonstrate high agreement with little to no systematic error and bias. Ongoing evaluation of new RTRI kit lots is important to ensure high quality test performance. Rejecting 9% of kit lots highlight the importance of continuing to work with manufacturers to ensure consistent kit production and quality assurance (QA) activities. Investing in effective QA measures, conducting both pre- and post-market performance data reviews, could help improve RTRI accuracy and outcomes in similar testing programs.

## Introduction

The 95-95-95 UNAIDS goals aim for 95% of all people living with HIV to be diagnosed, 95% of those diagnosed to start and be retained on antiretroviral therapy (ART), and 95% of those retained on ART to be virally suppressed by 2030 [1, 2]. Countries have looked for innovative measures to achieve the UNAIDS 95-95-95 goals and control the HIV epidemic. Some control measures are geared towards maximizing case finding such as community-based testing, index testing, self-testing, and laboratory testing [3, 4]. Use of incidence assays such as the lab-based Limiting Antigen Avidity EIA (LAg-EIA), in the cross-sectional studies can provide HIV incidence estimates and identify populations with ongoing transmission for effective public health intervention and epidemic control [5–7]. However, the application of these lab-based tests in the field has been limited for a variety of reasons, such as requiring highly skilled laboratory technicians, complicated equipment, additional supplies, and low temperature storage needed for test kits. Moreover, cross-sectional surveys are conducted infrequently due to high cost and logistical challenges and detect very few recent infections limiting further analysis [8]. The development of rapid tests for recent infection (RTRI) allows for rapid classification of HIV-1 infection as either a recent or long-term infection. When an RTRI testing algorithm is integrated into a robust case surveillance system, results can identify potential signals of HIV acquisition in geographic areas and populations and support the development of HIV recent infection surveillance [9–13]. Detection of recent HIV-1 infection occurs at point-of-testing or in the laboratory and allows countries to monitor the epidemic and respond appropriately with public health response measures to transmission hotspots [9, 14]. Successful implementation of TRACE begins with test kit performance to ensure high quality results. Although multiple factors can impact the quality of results, one of the first steps is to ensure kit lots are made with high consistency and are monitored using a standardized approach for quality control of test kits.

**Fig 1:**
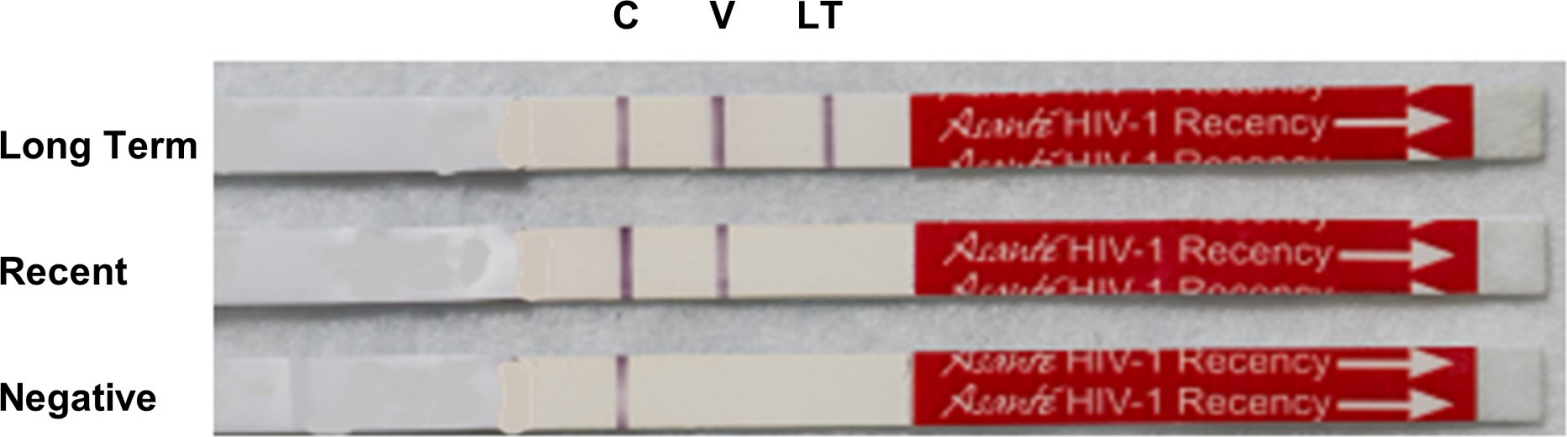
Schematic interpretation of the Asanté RTRI based on the absence or presence of the three lines; C = Control Line, V = Positive Verification Line, LT = Long Term Line.

The technology for RTRI was developed by CDC-ILB and was licensed to Sedia Biosciences. RTRI incorporates limiting amounts of a multi-subtype recombinant protein (rIDRM) on the long-term (LT) line to characterize HIV-1 infections as long-term or recent [5, 15]. In addition to the LT line, the HIV-positive verification (V) line detects HIV -1 and HIV-2 infection. This manuscript focuses on the Asanté HIV-1 Rapid Recency Assay marketed by Sedia Biosciences (Beaverton, OR) following licensing and successful transfer of technology from CDC. The Asanté RTRI is a lateral flow test with a control (C) line, V line, and LT line to detect HIV infection and differentiate between recent and long-term HIV-1 infection [16, 17]. The presence of only the C line indicates HIV-seronegativity [17]. The presence of all three lines (C line, V line, and LT line) indicates a long-term HIV-1 infection, whereas the absence of the LT line (in the presence of C line and V line) indicates a recent HIV-1 infection [17]. Fig 1 shows a visual interpretation of the Asanté RTRI assay. The assay has been evaluated by CDC-ILB and has a high diagnostic sensitivity (> 99%) and specificity (> 98%) and a high correlation with the Limiting Antigen-Avidity Enzyme Immunoassay (LAg-Avidity EIA) (Spearman’s Rank Correlation = 0.785) [16]. The NICD: National Institute for Communicable Diseases in South Africa has also evaluated the performance of the Asanté RTRI test with similar findings to those of CDC-ILB [18]. Field results and programmatic data from various countries implementing recent infection surveillance in PEPFAR programs have shown good performance in terms of sensitivity and specificity of the test and high agreement with various HIV national testing algorithms and/or with LAg-EIA [14, 19, 20]. However, Asanté tests are used for surveillance and not for HIV diagnosis or individual-level clinical management or care. WHO does not currently have a pathway for prequalification (PQ) assessment of any tests for recent infection surveillance. Programs should rely on their national HIV testing algorithm for HIV diagnosis.

Here, we describe the precise measures CDC-ILB has implemented to ensure that the Asanté RTRI kits are of high quality, and the statistical methods used to assess test kit performance and pass new kit lots prior to being on the market for end users.

## Materials and methods

**Fig 2:**
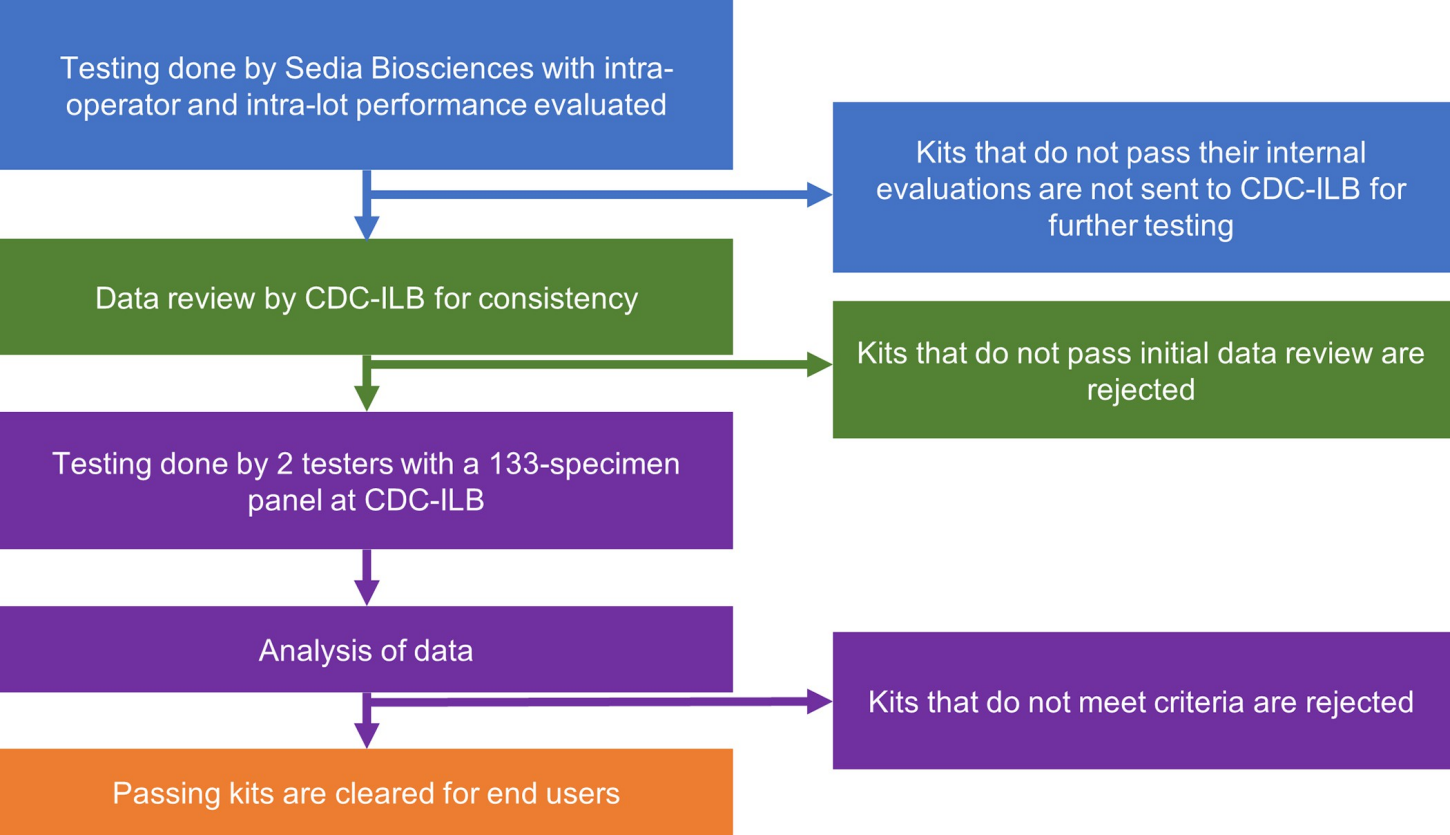
Schematic representation of the testing and analysis conducted by the manufacturer and CDC-ILB.

Kit lot evaluation is conducted both by the manufacturer and by CDC-ILB. Manufacturers conduct their own in-house quality control procedures. If a kit lot passes manufacturer review, test kits from the lot are then sent to CDC-ILB for additional quality evaluation. Fig 2 shows a schematic representation of the testing and analyses conducted by the manufacturer and CDC-ILB. It is important to note that the same set of 133 specimens were used for all evaluations conducted by both Sedia and CDC-ILB.

### Panel characterization and reference data

This evaluation uses a blinded well-characterized panel of 133 samples, consisting of five negative and 128 anti-HIV-1 positive samples that are a mix of recent and long-term infections covering the dynamic range of the intensity of the V and LT lines. Asanté test results are interpreted visually and then read with a reader supplied by the manufacturer [16]. This panel was characterized using the following reference tests: EIA + Western Blot for HIV diagnosis and Lag-EIA for recent infection classification. One of the 5 HIV-negative samples was negative by WB however it was consistently antibody positive for EIA and other rapid tests including the Asanté test (S1 Table provides additional details on the 133-panel with regards to reactivity levels and bands present). Over 100 aliquots of each sample were prepared with a maximum of five freeze-thaw cycles as well as 24/7 temperature monitoring to ensure sample integrity.

The initial Asanté kit lots were assessed against the reference test results to ensure successful technology transfer, while subsequent kit lot evaluations are compared against the mean of the first three lots that met both Sedia and CDC-ILB criteria. The criteria involved the following: 1) the positive verification line was compared against the EIA + WB algorithm and sensitivity/specificity were assessed (±1 sample misclassified for sensitivity and 100% specificity for all negative samples) and 2) the long-term line was compared against Lag-EIA using Pearson’s correlation coefficient using an agreement of > 0.90 as the passing criteria [21]. Additional criteria included inter-rater concordance at CDC-ILB. We performed a data analysis with the first 25 kit lots which passed quality control, and we did not observe any deviations in kit lot performance. We therefore calculated the mean reader values of the first 25 kit lots, which was used as the reference data for any subsequent kit lot evaluations. This mean was locked at 25 kit lots and is used as the reference data by both CDC-ILB and Sedia Biosciences for all current and ongoing kit lot evaluations including the evaluations described below. A locked mean is used to prevent moving averages and define control limits which can be used as reference for future kit lot comparisons.

### Kit lot quality control by sedia biosciences

The Asanté RTRI test kits evaluated in this study are manufactured by Sedia Biosciences Corp. (Beaverton, OR, USA). After every lot production and assembly of test kits, Sedia conducts in-house quality control testing which involves three independent testers using the same 133 panel of specimens that CDC-ILB uses, conducting linear regressions and 2x2 tables to compare the observed data (new lot) to the manufacturer’s reference data). Additional testing is performed by looking at the test kits from the beginning, middle, and end of manufacturing to compare intra-lot consistency. Prior to shipment of test kits, CDC-ILB receives the manufacturer’s data to conduct an initial data review. This involves interpretation of graphical and numerical data for evaluation of manufacturing consistency across the lot and comparison with prior manufacturer’s data of lots that passed qualification. If the data meets sensitivity, specificity, and consistent performance criteria, the test kits are then sent to CDC-ILB for additional testing. Any lot that does not pass this initial review is rejected, and no further testing is done.

### Testing at CDC-ILB

Test kits from lots that passed the initial data review were sent to CDC-ILB to undergo further evaluation at the CDC-ILB laboratory. After test kits were received, testing was conducted by two independent testers with the panel of 133 specimens, and at random intervals an additional 50 HIV-negative samples were also tested to assess specificity. Two results were recorded: band presence/absence were read visually, and then band intensity was quantified using the Asanté reader. Results were captured on a hard copy form then transcribed and analyzed electronically. A third tester ran the entire specimen panel when there were kit lot performance discrepancies between the results of the first and second tester within five days.

The Asanté reader is a hand-held instrument that quantifies the intensity of the C, V and LT lines in intensity units (IU). Samples with a V line IU ≥ 2.8 are classified as HIV-positive, while samples with a V line IU < 2.8 are HIV-negative [17]. If the LT line IU is ≥ 2.9 and the V line is ≥ 2.8, it is a long-term HIV-1 positive sample for all valid runs. If the LT line IU is < 2.9 and the V line is ≥ 2.8, it is a HIV-1 positive recent sample [17]. The slope and R^2^ of the reference vs. LT line and the reference vs. V line are calculated. To ensure consistency between the two testers, a linear regression was calculated, and evaluated against specific set criteria for slope and R^2^.

Using the following criteria of slope 1.00 ± 0.15 and R^2^ ≥ 0.9, linear regression analyses were conducted comparing the mean of the V and LT line reader data against the reference data. The LT line and V line data between operators were also analyzed for reproducibility using linear regression (0.85 ≤ slope ≤ 1.15 and R^2^ ≥ 0.90). Inter-rater reliability, using Cohen’s kappa with a cutoff of 0.85, was used to assess tests validity in this analysis. Data results from all kit lot evaluations are maintained in excel spreadsheets. The data are used to calculate the linear regressions.

### Bland-Altman analysis

For the purposes of this manuscript, we additionally conducted a Bland-Altman analysis of the data to assess the agreement between the first kit lot qualified and the mean of the subsequent passed 28 kit lots. We also assessed the agreement between the most recent kit lot (Kit Lot X) and the reference data. As an additional step, we analyzed the two individual testers’ results from Kit Lot X against each other. A Bland-Altman analysis was conducted to ensure there is no systematic error and/or bias, as well as assess tester and lot consistency [22, 23]. The plot of the difference against the mean allowed us to investigate any possible relationship between the measurement error and the true value [22, 23]. The true value was unknown and therefore the mean of the two measurements was the best estimated true value available [23]. Coefficient of variation was assessed by calculating the mean difference and the standard deviation. While some disagreement between results was expected, the level of disagreement is key in understanding whether the results from the kit lot under qualification were consistent with the reference data (i.e., biased or unbiased) [20, 21]. Given that the differences between results were normally distributed, 95% of the pairs of measurement are expected to be within two standard deviations of the mean difference. This assessment was performed to ensure an acceptable degree of discordance and to identify the existence of bias and/or systematic error when looking at the V and LT lines. SAS v9.4 was used to conduct the Bland-Altman analysis (SAS Institute, Cary, NC).

## Results

### Results from testing at CDC-ILB

**Fig 3:**
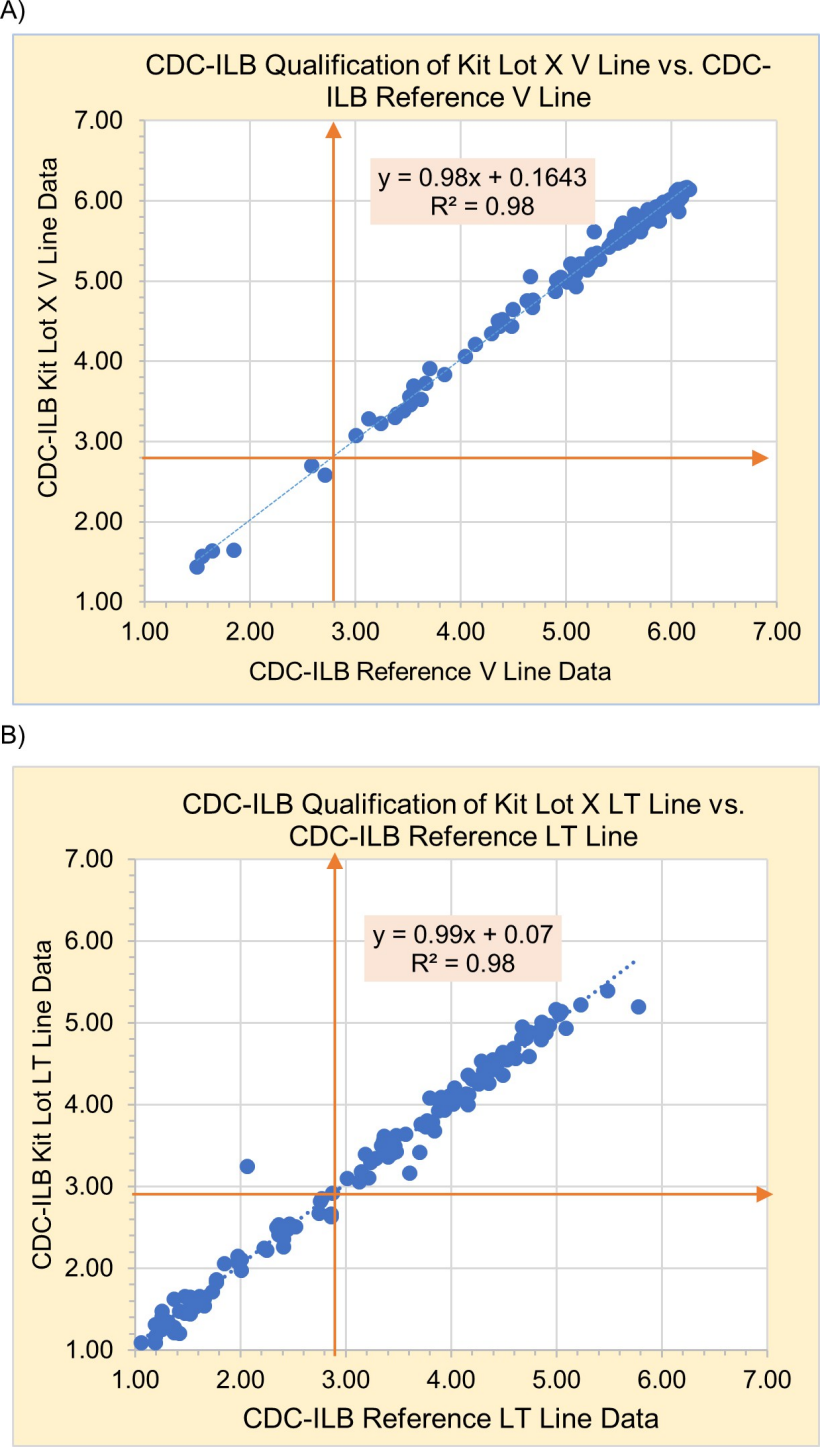
Regression analyses for the V and LT line of a kit lot against CDC-ILB reference data. A) Verification Line data for Kit Lot X against Reference Data. B) Long Term Line data for Kit Lot X against Reference Data. Orange arrows indicate cutoff values 2.8 IU for V line and 2.9 IU for LT lines.

From July 2017 to May 2020, 29 (90.6%) of 32 Asanté kit lots submitted by the manufacturer passed CDC-ILB’s additional evaluations. When looking at all 29 test kits that have met the defined performance criteria, the regression analyses demonstrate that test kits performed as expected with consistent R^2^ ≥ 0.92. On average, long-term specimens with IU ≥ 2.9, had a coefficient of variation <10%. However, for recent specimens (IU < 2.9 on the LT line) the average coefficient of variation was higher than 10%. An example of the regression analyses for the V and LT line has been provided in Fig 3, utilizing Kit Lot X, the most recent kit lot that passed our evaluation. Kit Lot X and the reference data have a high coefficient of determination (0.98), indicating that Kit Lot X data aligns with the reference data and have an expected positive R^2^ slope close to 1 (Fig 3). The overall relationship between the V line data for Kit Lot X against the reference data are as expected (Fig 3A). In Fig 3B, the overall relationship between the LT line data for Kit Lot X and the reference data are as expected with a high coefficient of determination (0.98) and a positive linear relationship. Overall, the 29 test kits had an average inter-rater reliability kappa of 0.9, indicating a strong level of agreement between testers.

### Bland-Altman analysis

**Fig 4:**
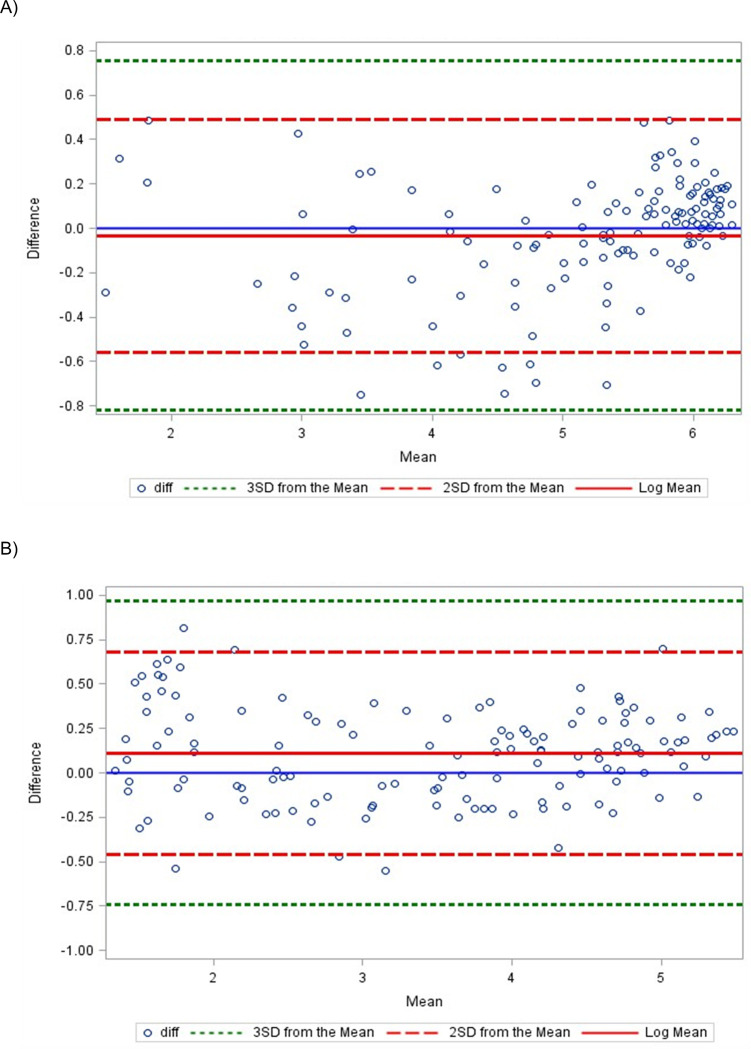
Bland-Altman Analysis Plot of Difference vs Mean comparing results from A) the V line of the first kit lot to the subsequent 28 and B) the LT line of the first kit lot to the subsequent 28.

The results comparing the first passed kit lot and the mean of the subsequent 28 passed kit lots demonstrate a high level of agreement with no systematic errors nor bias for the V and LT lines (Fig 4). Points were scattered evenly around the mean, despite a few outliers, and the narrow and reasonable limits. As the mean increased, there was no visible pattern, such as an increased slope or points that started out narrow then scattered widely. Additionally, the correlation between the first kit lot and the mean of the subsequent 28 lots for the V and LT line were low, with correlation coefficient of 0.30 and 0.05, both of which are below the standard cutoff of 0.70 [24].

**Fig 5:**
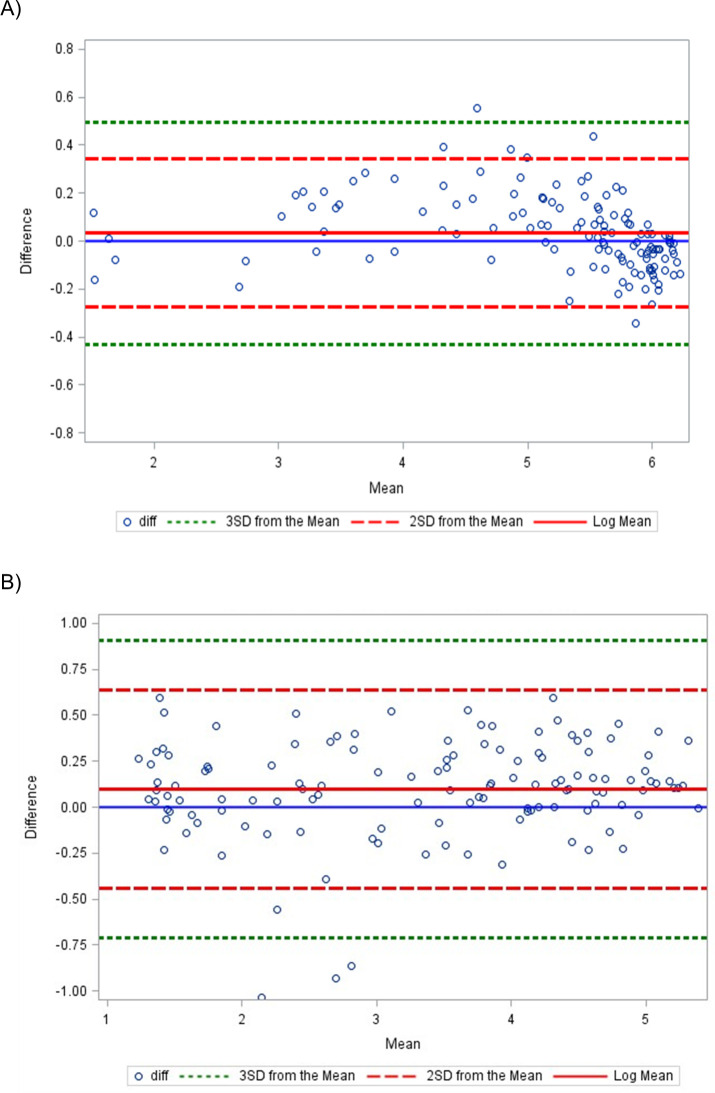
Bland-Altman Analysis Plot of Difference vs Mean comparing results from A) the V line of Kit Lot X to the reference data and B) the LT line of Kit Lot X to the reference data.

Results of the Bland-Altman analysis comparing the most recent Kit Lot X and the reference data indicate a high level of agreement (Fig 5). Points were scattered evenly around the mean, the majority of points were within 3 standard deviations of the mean, with limits that were narrow and reasonable. Similar to the results that compared the first lot to the subsequent 28 lots, there was no visible pattern on the graph and correlation was low for the V and LT lines (correlation coefficient of 0.45 and 0.13, respectively). Results from comparing the two testers indicated that there are no operator issues (S1 Fig). While the limits for the data between the testers for the LT line were larger due to the outliers noted earlier, overall results demonstrated a high level of agreement with no systematic error, minimal variance, agreement near 0, and no visible pattern in the scatter plot (S1 Fig).

## Discussion

This paper highlights the robust evaluation process undertaken by both the manufacturer and CDC-ILB to ensure that RTRI test kits are of the highest quality, given that previous studies have found that HIV rapid tests can vary in quality and result despite being WHO prequalified for market use [25–27]. To ensure proper transfer of technology, as well as successful use of RTRI in a programmatic surveillance setting, CDC-ILB’s additional robust quality assurance measures, beyond that of the manufacturer’s validations, ensures proper kit performance prior to market distribution. These extra steps are necessary to filter out poor performing lots that may be overlooked by the manufacturer. The need for consistency is even more critical for tests that distinguish between recent and long-term infection given the rarity of recent infections and its impact on recent infection classification.

Given that CDC-ILB failed three kit lots that initially met manufacturing criteria, our results highlight the importance of working with manufacturers to ensure consistent production of high-quality tests and reproducibility of test results across different laboratories. Following every kit lot evaluation, a report is sent to the manufacturer to inform whether the kit lot passed the evaluation, with the results of the performance evaluation conducted, as well as the criteria for pass/fail. Any failed kit lots are discarded by the manufacturer and a new kit lot is manufactured. A robust and continuous evaluation system for identifying poorly performing kit lots is necessary to ensure both proper performance and accuracy of recency results for successful implementation of TRACE. By conducting inter-laboratory and intra-lot comparisons, as well as monitoring historical performance of the test, these additional measures increase confidence in the ability of the test to perform as intended. Kit lots that failed either during CDC-ILB initial review of Sedia’s data or during testing at CDC-ILB have been attributed to many reasons ranging from sub-optimal conjugate concentration to inconsistent coating of protein antigen on the strips with striping of the antigen being the most common reason for failure. Results from the Bland-Altman analysis emphasize the consistency of the results, as there are no systematic errors nor significant discordance with the data for all 29 passed kit lots.

However, our evaluation of kit lots has a few limitations. Our 133-specimen panel does not include HIV-2 samples since the test is designed for only HIV-1 infections. Therefore, it is important that HIV-2 specimens are excluded from testing as they are likely to be reported as recent infection. Current implementation guidelines for the Asanté RTRI emphasize that recency testing should only be conducted on newly diagnosed HIV-1 infections to reduce potential misclassification from HIV-2, which is also captured by the Asanté RTRI product insert [17]. Another limitation is that both assessments, the manufacturer and CDC-ILB, used only plasma samples in the evaluation. Whole blood and serum are also acceptable sample types. As fingerstick samples are commonly used in many countries, CDC-ILB routinely monitors program data for any issues. To date, no major issue has been reported about the test performance in PEPFAR countries.

CDC-ILB will continue to conduct and provide this additional robust quality assurance activity to ensure proper test performance prior to distribution to countries. Countries are strongly encouraged to adopt robust temperature monitoring systems and quality assurance programs and evaluations upon delivery and before distribution prior to field use. Continuous review of post market surveillance and programmatic data can provide information on the continued acceptability of the test or removal of the product from the market, if needed. Additional investment in effective quality assurance measures should be considered when implementing RTRIs to minimize testing error in the field. Facilities should also implement the same quality assurance practices stated above as well as conduct routine quality control testing. Aside from quality assurance prior to deployment, it is essential for testers to receive proper training to ensure tester confidence and accuracy to achieve high level of testing accuracy. Therefore, for the Asante RTRI hands-on training currently involves 13 well characterized specimens that include a mix of recent, long-term, and HIV-negative infections. Testers who pass this hands-on practical exam and the written exam are then certified as RTRI testers [3]. RTRI tests could be integrated into routine HIV proficiency testing to assess tester competence and process management procedures. These are a few of these additional processes currently implemented as part of the on-going quality initiatives for the TRACE program. All these processes are essential to achieving the highest level of accuracy and reliability of test results. Two additional manufacturers have licensed the technology and will go through similar evaluation procedures and kit lot qualification criteria for use of their RTRI tests in PEPFAR programs or beyond.

## Supporting information

S1 Fig Bland-Altman Analysis Plot of Difference vs Mean comparing results from A) the V line of Tester 2 to Tester 1 and B) the LT line of Tester 2 to Tester 1.
(TIF)

S1 Table Characteristics of the 133-specimenal panel. (XLSX)

S1 Data (XLSX)
