# Supplementary figures and images for: Continuous quality evaluation of the Asanté rapid test for recent infection for robust kit lot quality verification
Source: PLOS Glob Public Health. 2024 May 14;4(5):e0003195. doi: 10.1371/journal.pgph.0003195 (PMC11093390; doi:10.1371/journal.pgph.0003195)

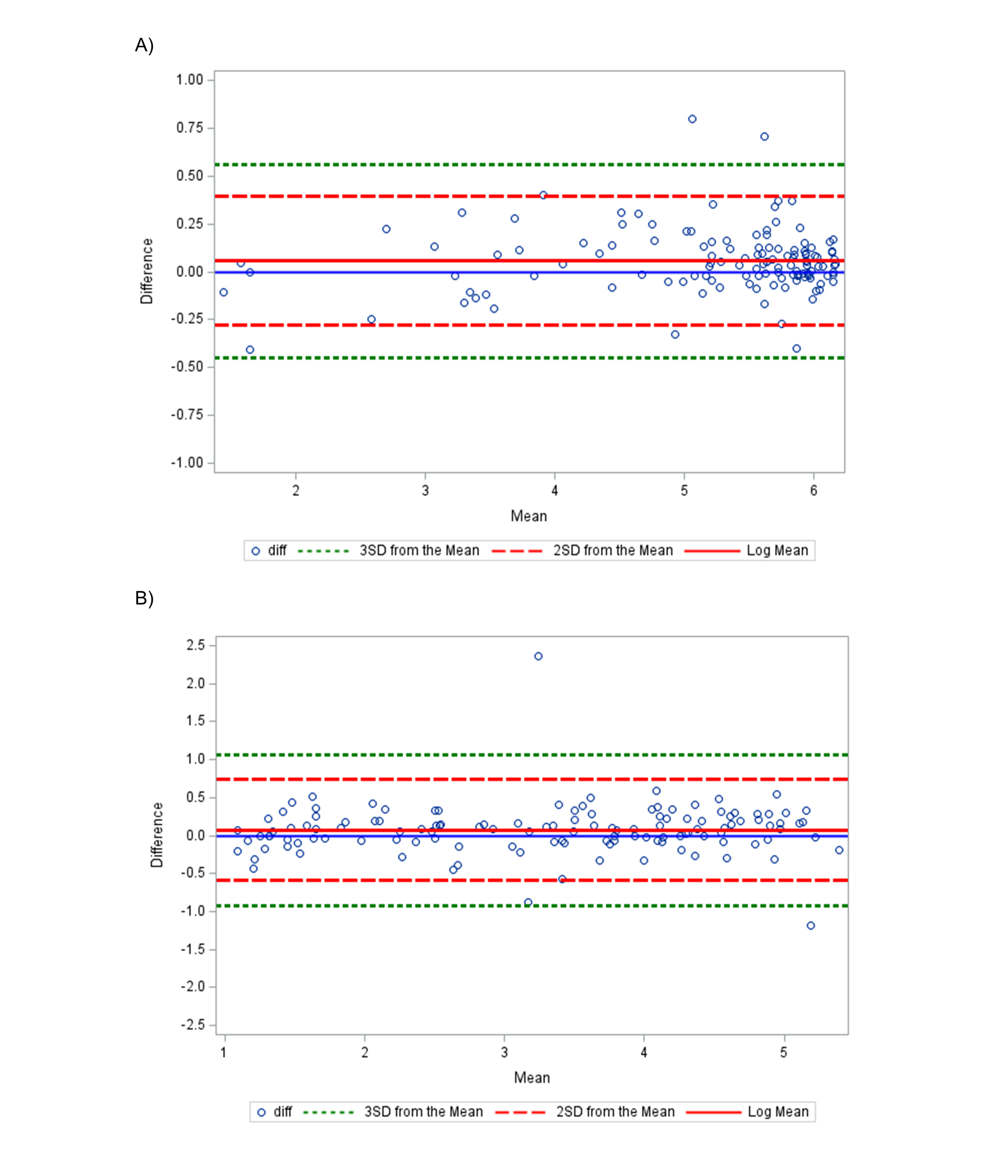

Supplement: S1 Fig — Bland-Altman Analysis Plot of Difference vs Mean comparing results from A) the V line of Tester 2 to Tester 1 and B) the LT line of Tester 2 to Tester 1. (TIF) [file pgph.0003195.s001.tif]
